# The HHV-6B U20 glycoprotein binds ULBP1, masking it from recognition by NKG2D and interfering with natural killer cell activation

**DOI:** 10.3389/fimmu.2024.1363156

**Published:** 2024-06-17

**Authors:** Grant C. Weaver, Christine L. Schneider, Aniuska Becerra-Artiles, Kiera L. Clayton, Amy W. Hudson, Lawrence J. Stern

**Affiliations:** ^1^ Immunology and Microbiology Graduate Program, Morningside Graduate School of Biomedical Sciences, UMass Chan Medical School, Worcester, MA, United States; ^2^ Department of Pathology, UMass Chan Medical School, Worcester, MA, United States; ^3^ Department of Microbiology and Immunology, Medical College of Wisconsin, Milwaukee, WI, United States; ^4^ Department of Biochemistry and Molecular Biotechnology, UMass Chan Medical School, Worcester, MA, United States

**Keywords:** immune evasion, human herpesvirus, major histocompatibility complex, stress receptor, natural killer cell ligand, surface masking, small-angle X-ray scattering

## Abstract

**Introduction:**

Human Herpesvirus 6B (HHV-6B) impedes host immune responses by downregulating class I MHC molecules (MHC-I), hindering antigen presentation to CD8+ T cells. Downregulation of MHC-I disengages inhibitory receptors on natural killer (NK) cells, resulting in activation and killing of the target cell if NK cell activating receptors such as NKG2D have engaged stress ligands upregulated on the target cells. Previous work has shown that HHV-6B downregulates three MHC-like stress ligands MICB, ULBP1, and ULBP3, which are recognized by NKG2D. The U20 glycoprotein of the related virus HHV-6A has been implicated in the downregulation of ULBP1, but the precise mechanism remains undetermined.

**Methods:**

We set out to investigate the role of HHV-6B U20 in modulating NK cell activity. We used HHV-6B U20 expressed as a recombinant protein or transduced into target cells, as well as HHV-6B infection, to investigate binding interactions with NK cell ligands and receptors and to assess effects on NK cell activation. Small-angle X-ray scattering was used to align molecular models derived from machine-learning approaches.

**Results:**

We demonstrate that U20 binds directly to ULBP1 with sub-micromolar affinity. Transduction of U20 decreases NKG2D binding to ULBP1 at the cell surface but does not decrease ULBP1 protein levels, either at the cell surface or in toto. HHV-6B infection and soluble U20 have the same effect. Transduction of U20 blocks NK cell activation in response to cell-surface ULBP1. Structural modeling of the U20 – ULBP1 complex indicates some similarities to the m152-RAE1γ complex.

## Introduction

Human Herpesvirus 6B (HHV-6B) infects over 90% of the world’s population by the age of 6 (1, 2). To facilitate lifelong latency, it is necessary for HHV-6B to modulate the host immune response. However, unlike fellow β-herpesvirus family member human cytomegalovirus (HCMV), these immune evasion strategies remain poorly characterized for the roseoloviruses, particularly HHV-6B. None of the immune evasion genes expressed by HCMV are conserved in HHV-6B. However, the HHV-6B genome contains a block of six genes (U20-U26) that are unique to the roseolovirus genus, which could be where the “missing” immune evasion genes might reside.

Previous studies have shown that viral homologues of nonclassical MHCI proteins are a common tool used by herpesviruses and poxviruses to modulate host immunity (reviewed in (3)). Structural modeling strongly suggests that the HHV-6B U20 and U21 glycoproteins are virally-encoded Major Histocompatibility Complex protein homologs (vMHCs) and that U20 and U21 are the only predicted vMHCs in the U20-U26 gene cluster (3). One common immune evasion strategy utilized by herpesviruses is downregulation of classical class I MHC (MHC-Ia) proteins to prevent presentation of viral antigens to CD8^+^ T cells. In roseoloviruses, including HHV-6B, the U21 glycoprotein achieves this by binding directly to MHCs and redirecting them to the lysosome for degradation (4–6). However, since this loss of MHCI risks triggering NK cell mediated “missing self” recognition and activation, the virus must also counter this response by manipulating activating and inhibitory NK receptors or their ligands.

The MIC/ULBP family of proteins are non-classical MHC class I (MHC-Ib) molecules that are upregulated under various stress conditions such as heat shock, tumor transformation, or viral infection (reviewed in (7, 8)). HHV-6B infection results in downregulation of MICB, ULBP1, and ULBP3 (9). Since it was recently shown that U20 glycoprotein downregulates ULBP1 in HHV-6A infection (10), we hypothesized that U20 may have a similar function for HHV-6B. In this study, we demonstrate that HHV-6B U20 binds directly to ULBP1. Furthermore, both cellular and soluble U20 can inhibit NKG2D binding to ULBP1-expressing cells, which blocks NKG2D-mediated NK cell activation. Structural modeling of the U20-ULBP1 complex guided by small-angle X-ray scattering (SAXS) data show that the expected binding site on ULBP1 for NKG2D is occluded in the complex. Together, these findings suggest that U20 helps protect infected cells from NK cell surveillance by masking ULBP1 from detection by NKG2D.

## Materials and methods

### Cell culture and cell line preparation

The full coding sequences of ULBP1 (Uniprot Q9BZM6) and HHV-6B U20 (Uniprot Q9QJ46) were synthesized by Genscript and cloned into the pCDH lentiviral vector. To aid in selecting transduced cells, the ULBP1 plasmid contained a puromycin resistance cassette and the U20 plasmid contained an IRES-GFP reporter. Lentivirus was generated by transfecting 293T cells and collecting supernatant as previously described (11). First, a ULBP1 expressing cell line was prepared by culturing HEK293S GnTI cells (obtained from ATCC, catalog number CRL-3022). in the presence of various dilutions of the ULBP1 lentivirus, followed by selection in medium containing 4 μg/mL puromycin. To ensure expression of ULBP1, the cells were sorted by anti-ULBP1 staining at the UMass Chan Medical School Flow Cytometry Core. To ensure consistent ULBP1 expression, these cells were used as the basis for the double transduced cells (ULBP1/U20). ULBP1 expressing cells were incubated with U20-GFP lentivirus, expanded, and sorted for GFP expression. An identical procedure was carried out to generate U20 and ULBP1 expressing cell lines with fully intact glycans using HEK293T cells, also obtained from ATCC (CRL-3216).

U373 cells stably expressing either ULBP1 or an N terminally HA-tagged ULBP1 were generated by retroviral transduction using the vector pLNCX (Takara). These cell lines were then transduced with a lentiviral vector (pHAGE-puro-MCS (pPM-U20), in which U20 expression was controlled by the CMV promoter and an IRES-driven puromycin N-acetyl transferase gene (*Pac*) allowed for puromycin selection of transductants (12).

### Protein production and purification

Soluble U20 extracellular domain (sU20) was prepared by growing stably transduced HEK293 GnTI cells. These cells do not express N-acetylglucosaminyltransferase I (13), enabling almost complete deglycosylation by Endoglycosidase H and improving homogeneity of purified protein for downstream biochemical assays. Transduced cells were grown in Freestyle 293 Expression Medium (Thermo Fisher Scientific) for 1–2 weeks with harvest/media exchange occurring every 2–3 days. The resulting supernatant was purified by Nickel-NTA affinity purification, followed by Cation Exchange Chromatography and a final size exclusion step using a Superdex 200 Increase 10/300 GL column (Cytiva). In some cases, buffer-exchanged nickel eluate was treated with EndoH (NEB) to remove the high mannose glycans. For calculations of molecular weight of EndoH treated proteins, we added the 221 Da weight of the single remaining GlcNAc at each site. For glyco-intact proteins produced in HEK293S GnTI cells, we assumed an average of seven mannose residues per glycan and added an additional 1.56 kDa to the predicted mass for each glycosylation site.

The ULBP1–6xHIS soluble construct (ULBP1–6H) was designed by adding a single glycine spacer and a 6xHIS tag to the C-terminus of the protein. ULBP1-Fc was designed by adding a Factor Xa cleavage site to the 3’ end of the coding sequence, followed by the Fc domain of human IgG1 (Uniprot P0DOX5, P228-K449), and a BirA recognition sequence for site specific biotinylation (Avidity). We made a number of modifications to the IgG sequence to abolish binding to Fc receptors. Substitutions were made at what would be positions 327, 330, and 331 in a complete IgG1 heavy chain to match IgG4 (14, 15).

ULBP1–6H and ULBP1-Fc were prepared by transient transfection of Expi293 cells. For 6xHIS ULBP1, the resulting supernatant was purified on a Nickel-NTA column followed by size exclusion chromatography. For ULBP1-Fc, the supernatant was run over a protein A column followed by size exclusion chromatography on a Superdex 200 Increase 10/300 GL column (Cytiva). Additionally, ULBP1-Fc was biotinylated for SPR studies using the BirA site specific biotinylation kit (Avidity).

### Glycan characterization

Soluble U20 extracellular domain (sU20) was deglycosylated using either EndoH or PNGaseF (NEB) according to the manufacturer’s instructions. Following deglycosylation, 5 μg of protein was subjected to reducing SDS-PAGE and the gel was stained with GelCode Blue (Thermo). Bands containing U20 were cut out and submitted to the UMass Chan Mass Spectrometry Facility for further processing. The protein was subjected to in-gel digestion with trypsin and/or GluC and analyzed on an Orbitrap Fusion Lumos Tribrid Mass Spectrometer. Glycosylation sites were identified by searching for modified peptides. EndoH digested glycans leave behind a single GlcNAc, which adds 221 daltons to the expected size of the peptide while PNGaseF treated glycans result in an asparagine to aspartic acid substitution.

### Surface plasmon resonance assays

The NKG2D ligand studies were performed as follows. On a Biacore 3000 instrument (Cytiva), biotinylated NKG2D-Fc, MICB-Fc, and ULBP3-Fc (R&D Systems) as well as biotinylated ULBP1-Fc (made in house) were coupled to a Neutravidin coated CM5 chip. Approximately 2000 response units of each protein was immobilized. Following ligand immobilization, 10 μL of sU20 was injected at a rate of 5 μL per minute at concentrations ranging from 0.125 μM to 16 μM. The maximum response for each concentration was plotted using GraphPad Prism and fitted using a one site, specific binding model to estimate an apparent equilibrium dissociation constant.

The affinity of U20 for ULBP1 was measured similarly. Biotinylated ULBP1-Fc was coupled to a Neutravidin coated CM5 chip in amounts ranging from 1000–2500 response units and U20 was injected at a rate of 5 μL per minute at concentrations ranging from 0.125 μM to 32 μM. A biotinylated CD200 receptor Fc fusion protein was also coupled to the chip as a negative control and the signal from this flow cell was subtracted from the other traces. Dissociation constants were determined as for NKG2D.

### Co-immunoprecipitation of U20 and HA-ULBP1

U373 cells expressing an N-terminally HA-tagged ULBP1 and either U20 or the empty vector were washed with PBS then lysed in digitonin lysis buffer (1% digitonin, 150 mM NaCl, 50 mM Tris-HCl [pH 7.4], 5 mM N-ethyl-maleimide (NEM), 0.1 mM phenylmethylsufonyl fluoride (PMSF) for 5 min at 37°C to solubilize lipid rafts. Lysates were centrifuged for 10 min at 16,000 X g at 4°C to pellet nuclei and debris. Lysates were normalized to total protein concentration (320 µg per immunoprecipitation) as determined by BCA assay (Thermo Fisher). Lysates were then incubated with either a rabbit polycolonal antibody to U20 (MCW53) and protein A agarose or a monoclonal antibody to the HA tag (HA.11(Biolegend)) and protein G agarose for 4 hours at 4°C. Immunoprecipitates were washed four times with digitonin wash buffer (0.1% digitonin, 150 mM NaCl, 50 mM Tris [pH 7.4]), subjected to reducing SDS-PAGE, transferred to BA-85 nitrocellulose membrane (Whatman) and probed with designated primary antibodies followed by an appropriate HRP conjugated secondary antibody (BioRad). Bands were visualized using SuperSignal reagent (Thermo Fisher) and quantified with an Alpha Imager (AlphaInnotech, San Leandro, CA).

### Pulse chase analysis of ULBP1

Cells were detached with trypsin and incubated in methionine- and cysteine-free DMEM (Invitrogen) supplemented with 2% FBS for 30 min at 37°C to deplete endogenous Met and Cys. The cells were then labeled with 700 µCi/ml of [^35^S]-Express label (1100 Ci/mmol; PerkinElmer) for 15 minutes at 37°C and chased with complete DMEM supplemented with 1 mM non-radioactive methionine and cysteine for indicated times at 37°C. When indicated, lysosomal inhibitors leupeptin (Sigma) and folimycin (EMD, San Diego, CA) were added at 200 µM and 20 nM, respectively, during the starve, pulse, and chase. Cells were washed with PBS then lysed in Triton X-100 lysis buffer (10 mM Tris-HCl [pH 7.4], 150 mM NaCl, 1% Triton X-100, 0.1 mM PMSF and 5 mM NEM for 5 min at 37°C, followed by rocking for 10 min at 4°C. Lysates were centrifuged for 10 min at 16,000 X g at 4°C to pellet nuclei and debris. Clarified lysates were incubated overnight at 4°C with a ULBP1 antibody (Clone m295, Amgen) and protein G agarose (Invitrogen). Immunoprecipitates were washed four times with Triton X-100 wash buffer (10 mM Tris-HCl [pH 7.4], 150mM NaCl, 1% Triton X-100), and subjected to SDS-PAGE gel electrophoresis.

### ULBP1 immunoblots

Equal numbers of HEK293S GnTI, ULBP1, and ULBP1+U20 transduced cells were harvested and lysed in 1% NP-40 lysis buffer with protease inhibitors (Sigma). The resulting protein was run on a 4–20% gradient gel (Biorad) and transferred to a PVDF membrane. After blocking, ULBP1 was detected with an anti-ULBP1 primary (Abcam, ab176566) and an anti-rabbit secondary (Abcam, ab205718) with anti-tubulin (Abcam, ab40742) as a loading control. The blots were visualized on a Biorad GelDoc Go imaging system.

U373 cells expressing ULBP1 with or without U20 were treated with lysosomal inhibitors 200 µM leupeptin and 20 nM folimycin for 16 hours prior to lysing in RIPA buffer (50 mM Tris HCl [pH 7.4], 150 mM NaCl, 1% Triton X-100, 1% sodium deoxycholate, 0.1% SDS, 1 mM EDTA) supplemented with 5 mM NEM and 0.1 mM PMSF. Lysates were normalized to total protein (10 µg per lane) by BCA assay, analyzed by SDS-PAGE and immunoblotting as describe above for the co-IP experiment.

### NKG2D-Fc binding assays

For membrane bound (cellular) U20: WT, ULBP1, and ULBP1/U20 HEK293S cells were detached using Versene (ThermoFisher Scientific) to preserve the integrity of membrane proteins. These cells were then counted and 1x10^5^ cells were added to a 96-well V-bottom plate. The cells were then washed and 5 nM of NKG2D-Fc (R&D Systems) was added along with 2 μL of AlexaFluor647-conjugated Anti-Human IgG F(ab’)_2_ (Jackson ImmunoResearch), and incubated on ice for 30 minutes. Subsequently, the cells were washed twice and analyzed using an LSRII instrument with FACSDiva software (BD Biosciences). All data were analyzed using FlowJo 10.6.0 software (FlowJo, LLC).

For soluble U20: WT and ULBP1 HEK293S cells were detached, counted, and plated as above, except that before staining they were pretreated with sU20 at various concentrations for 5 minutes on ice. Following the pretreatment, they were stained and analyzed as described above.

### HHV-6B infection

The human T lymphoblast cell line SupT1 (CRL-1942, American Type Culture Collection, Manassas, VA) was used for infection with HHV-6B strain Z29 as described previously (16). Briefly, cells were incubated with virus-containing supernatant of HHV-6B-infected SupT1 cells (300 copies of viral DNA per cell) at 37°C. Supernatant collected from uninfected SupT1 was used for mock infections. After 3 hours, cells were centrifuged, the supernatant discarded, and cells washed with phosphate buffered saline, pH 7.4 (PBS). Finally, cells were resuspended in RPMI 1640 medium supplemented with L-glutamine, penicillin/streptomycin, and 5% fetal bovine serum (FBS) at a density of 500,000 cells/mL and transferred to 6 well plates. Infected cells were collected at 48 and 72 hours for western blotting and flow cytometry respectively. Infection was verified by RT-PCR targeting multiple viral genes (detailed in **Supplementary Information**) and flow cytometry staining for the gB glycoprotein, using an antibody provided by the HHV-6 Foundation.

### NK cell isolation and recognition assays

PBMCs were isolated from Leukochambers (Rhode Island Blood Center) using Lymphoprep media and SepMate tubes (StemCell Technologies) per the manufacturer’s instructions. PBMCs were then frozen down in 10% DMSO in FBS and stored in liquid nitrogen until needed.

NK cells were prepared as described previously (17). Briefly, healthy donor PBMCs were thawed and rested for two nights in RPMI containing 50 ng/mL IL-15 (Biolegend). On the third day, NK cells were isolated using a CD56 positive selection kit (StemCell Technologies). Following isolation, the NK cells were labeled with CellTrace Violet. The NK cells (50,000 or 100,000 per well) were then cocultured with Jurkat cells expressing U20 and/or ULBP1 for six hours in the presence of anti-CD107a-Alexa Fluor 488 (Biolegend, 10 ng/mL IL-2, and GolgiStop/GolgiPlug (BD). Unless otherwise shown the ratio of effector NK cells to target Jurkat cells was 1:1. After the six-hour stimulation, the NK cells were stained for surface markers (**Supplementary Table S2**), fixed/permeabilized, stained for interferon-gamma (IFNγ) (**Supplementary Table S2**), and subjected to analysis by flow cytometry. The data were analyzed using FlowJo 10.8. Based on positive and negative controls, gates were drawn for IFNγ and CD107a positivity in each donor replicate and we reported the median fluorescence intensity for each marker. Background activation was accounted for by subtracting the signal from the uncoated well. Each of the three donors were tested a total of six times.

### Size exclusion and size exclusion/multi-angle light scattering studies of U20-ULBP1 complexes

Equimolar amounts of ULBP1–6H and U20 or ULBP1-Fc were mixed and incubated at room temperature for 1 hour and then subjected to size exclusion chromatography. The ULBP1-Fc complex was run over a Superose 6 Increase 10/300 GL column (Cytiva) and the ULBP1–6H complex was run over a Superdex 200 Increase 10/300 GL column (Cytiva). The fractions were subjected to reducing SDS-PAGE and the bands were visualized by staining with GelCode Blue (Pierce).

Size exclusion/multi-angle light scattering (SEC-MALS) was performed at room temperature using a 1260 Infinity HPLC system (Agilent), an Optilab T-rEX differential refractive index detector (Wyatt Technology), and a Dawn Helios-II multi-angle light scattering detector (Wyatt Technology). Sample concentration was adjusted to 1 mg/ml with gel filtration buffer (PBS) and passed through a 0.22 μm filter to remove any aggregates or debris. 50 μl of sample was injected onto a WTC-030S5 size exclusion column (Wyatt Technology) pre-equilibrated with gel filtration buffer. Data analysis was performed using Astra 6 software (Wyatt Technology).

### Computational structure prediction for U20 and ULBP1

Structural models for U20 were generated using various methods, specifically AlphaFold2 via ColabFold, RoseTTAFold, and ESMfold (18–20). Since no single model had a high overall confidence score, we separated each predicted structure into MHC platform and Ig domains and tested different combinations of domains derived from each prediction tool. This also allowed for sampling of a broad range of interdomain angles, as this parameter was predicted with a very high degree of variance across the different prediction tools.

### Size exclusion/small angle x-ray scattering

SEC-SAXS was performed at NSLS-II using their mail-in service at the Life Sciences X-ray Scattering (LiX) 16-ID beamline (21–23). EndoH treated U20, ULBP1–6H, and ULBP1-Fc as well as equimolar mixtures of U20 and ULBP1–6H or ULBP1-Fc were concentrated to 4.5mg/mL and shipped on ice. Upon arrival at the beamline, the samples were filtered using 0.2um nylon SpinX tubes (Corning) and passed over a Superdex 200 Increase 5/150 GL column (Cytiva) at 0.5 mL per minute on an HPLC system for in line SAXS measurements. Buffer subtraction, peak selection, and profile analysis were performed with LiXtools (23). Ab initio reconstructions were performed using DAMMIF (24, 25). Twenty independent DAMMIF-derived bead models were then averaged using DAMAVER (26) to obtain a consensus molecular envelope. Hypothetical models of protein complexes were then input into Crysol (27) to generate theoretical scattering curves that were then compared against the experimental data.

### Generation of models for fitting to SAXS data

Models input to Crysol were generated using multiple methods. Some models were generated by automatically fitting predicted structures into the envelope volumes created from the bead models using Chimera (28). Other models were generated by manually manipulating the positions of predicted structures of the separated domains of U20 and/or ULBP1 to fit closely into the bead model derived envelopes. Still other models were generated by rotating and translating segments of the predicted structures at the amino acid level using Coot (29). Some or all of these methods were used in concert to optimize the chi square value of the Crysol fit of our models to the experimental scattering data.

## Results

### U20 binds to ULBP1 but not ULBP3 or MICB

**Figure 1:**
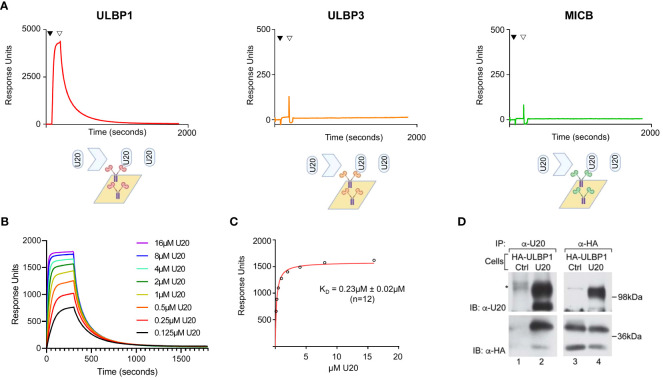
U20 binds directly to ULBP1 but not ULBP3 or MICB. **(A)** SPR analysis of U20 binding to NKG2D ligands. Fc fusions of ULBP1, ULBP3, and MICB were coupled to a neutravidin chip at equivalent levels and sU20 was injected at a concentration of 32 μM on a Biacore 3000 instrument. **(B)** Representative injection series for U20 flowed over ULBP1 in a twofold dilution series from 0.125 μM to 16 μM. **(C)** Representative equilibrium binding curve fit. K_D_s were calculated by nonlinear curve fitting using GraphPad Prism and the mean K_D_ and standard deviation are shown. **(D)** U373 cells expressing HA-ULBP1 and either U20 or a vector control were lysed in digitonin lysis buffer and immunoprecipitated using antibodies to U20 (MCW53) or HA (HA.11). Proteins were resolved by SDS-PAGE and detected by immunoblot using antibodies to U20 (MCW53) or HA (HA.11). Migration positions of molecular weight markers are indicated.

We began by evaluating whether U20 could participate in downregulation of the non-classical MHC-1b ligands ULBP1, ULBP3, or MICB through direct binding interactions. We used surface plasmon resonance assays (SPR) to measure binding of recombinant soluble U20 extracellular domain (sU20) to immobilized Fc-fusion proteins carrying the extracellular domains of the NKG2D ligands. U20 bound strongly to ULBP1, but no binding was detected for MICB or ULBP3 at concentrations up to 16 μM (**Figure 1A**). The concentration series of U20 binding to immobilized ULBP1-Fc was fit to a single-site specific binding model, resulting in an equilibrium dissociation constant K_D_ ~0.23 ± 0.02 μM at 20°C (**Figures 1B, C**). Dissociation kinetics were complex and could not be fit by a simple one-site binding model, with dissociation half-life varying from 63–124 seconds over the concentration range tested (**Figure 1B**). The binding of U20 to ULBP1 is ~4-fold stronger than the binding of ULBP1 to its natural ligand NGK2D, K_D_ ~1 μM [**Supplementary Figure S1A** and ref (30)].

To determine whether U20 and ULBP1 interact within a cell, we co-expressed U20 and an N-terminally HA tagged ULBP1 in U373 cells. When we immunoprecipitated using a U20 antibody, HA-ULBP1 was recovered (**Figure 1D**, lane 2, lower panel) and when we immunoprecipitated using an HA antibody, U20 was recovered (**Figure 1D**, lane 4, upper panel), confirming that these proteins interact within the cell. It is worthwhile to note that both U20 and HA-ULBP1 migrate as two bands in the immunoblots of the lysates (**Figure 1D**, lanes 2 upper panel, and lanes 3 and 4, lower panel) with the lower band being the newly synthesized protein that lacks complex glycosylation and post-translational modifications while the upper band represents the mature form of each protein. Interestingly, although both forms of each protein are present in the lysates, the interaction of these proteins appears to be skewed toward the mature forms of each protein (compare **Figure 1D**, upper panel, lane 2 vs 4 and lower panel, lane 4 vs 2).

### U20 does not increase lysosomal degradation or reduce the overall levels of ULBP1

**Figure 2:**
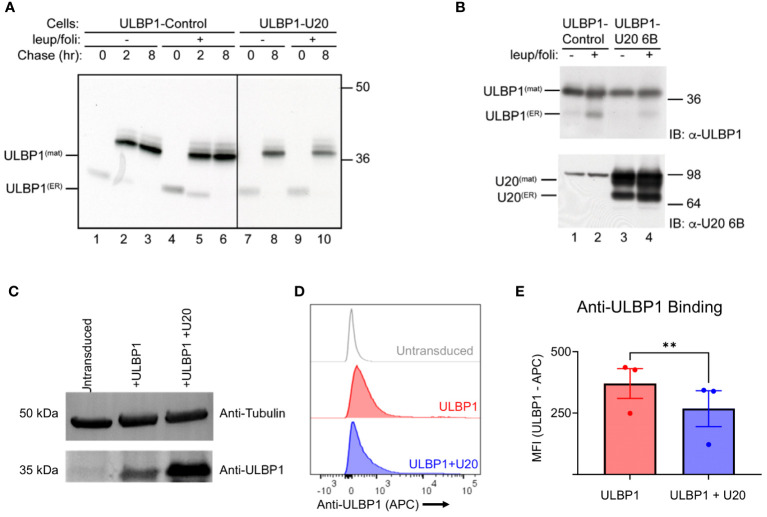
Cellular U20 decreases surface staining of ULBP1 without decreasing total ULBP1 levels. **(A)** Cells were pulse labeled for 30 minutes with 35S-methionine, chased for 0, 2 or 8 hours, then lysed in Triton X-100 lysis buffer supplemented with 5 mM NEM and 0.1 mM PMSF. When indicated, cells were incubated in leupeptin (200 μM) and folimycin (20 nM). ULBP1 was immunoprecipitated using α-ULBP1 (m295) and analyzed by SDS-PAGE and autoradiography. Migration positions of the immature (ULBP1(ER)) and mature (ULBP1(mat)) forms of ULBP1 and molecular weight markers are indicated. **(B)** Cells were treated with 200 µM leupeptin and 20 mM folimycin for 16 hours prior to lysing in RIPA buffer supplemented with 5 mM NEM and 0.1 mM PMSF. Cell lysates (10 µg) were immunoblotted using antibodies to ULBP1 (AF1380) or U20 (MCW52). **(C)** Anti-ULBP1 western blot (Top) and anti-tubulin western blot (bottom). **(D)** Representative flow cytometry data showing level of anti-ULBP1 binding to HEK293 cells transduced with ULBP1 ± U20. **(E)** MFIs were plotted for three independent anti-ULBP1 staining experiments. The mean ± SD is shown. Statistical analysis for paired t test, **p < 0.01.

One common mechanism of viral interference with NK activating ligands is through degradation or intracellular sequestration that prevents surface expression (3). To assess whether U20 affects lysosome-mediated turnover of ULBP1, we performed pulse-chase experiments in the presence of the lysosomal protease inhibitor leupeptin and the vacuolar H+-ATPase inhibitor folimycin. Stabilization of a protein by lysosomal protease inhibitors would suggest a role for lysosomal proteases in the turnover of that protein. In U373 cells stably expressing ULBP1 but lacking U20, ULBP1 migrated at approximately 31 kDa after a 30-minute pulse label, which is converted to a more slowly migrating ~37 kDa polypeptide by the 2-hour chase point (**Figure 2A**, lanes 1–3). This is consistent with our previous pulse chase results with the increase in ULBP1 size at later chase points due to either N- or possibly O-linked glycan modifications (31). Similarly, as we demonstrated previously, we recovered less labeled ULBP1 immediately following the pulse (0-hour chase) than at later chase periods (**Figure 2A**, lane 1 vs 2 or 3) possibly due to differences in recognition by the monoclonal antibody used to precipitate ULBP1 (31). In cells lacking U20, ULBP1 was stable throughout the 8-hour chase and the addition of lysosomal inhibitors did not appear to increase its stability (**Figure 2A**, lanes 1–3 vs 4–6). In cells expressing U20, ULBP1 was also stable throughout the 8-hour chase and lysosomal inhibitors did not increase ULBP1 stability (**Figure 2A**, lanes 7 and 8 vs 9 and 10). These results suggest that U20 does not increase lysosomal-mediated turnover of newly synthesized ULBP1.

Having established that U20 did not increase lysosomal degradation of newly synthesized ULBP1 we also wanted to determine whether U20 might affect the steady state levels of ULBP1. In U373 cells expressing ULBP1, but lacking U20, the predominant form of ULBP1 was the mature form (**Figure 2B**, lane 1 upper panel). The addition of lysosomal inhibitors increased the amount of the immature form but did not affect the level of the mature form (**Figure 2B** lane 1 vs 2 upper panel). In ULBP1-U373 cells also expressing U20, the findings were similar, suggesting U20 does not promote degradation of steady state ULBP1 (**Figure 2B**, lane 3 vs 1 and 4 vs 2, upper panel).

Given that U20 did not appear to destabilize ULBP1, we next examined whether it affected the amount of ULBP1 on the cell surface. To do this, we used lentiviral vectors to stably transduce HEK293S GnTI cells with full length U20 and/or ULBP1. First, we lysed the U20-expressing HEK293 cells and performed an anti-ULBP1 western blot to examine steady-state levels of ULBP1 and confirm that the behavior of this system was consistent with our observations in U373 cells (**Figure 2C**). The U20-transduced cells expressed somewhat more ULBP1 than the ULBP1-transduced cell line from which they were made (**Figure 2C**). Interestingly, we observed lower levels of ULBP1 surface staining in the U20-transduced cells (**Figures 2D, E**), despite the increase in total protein levels that we observed by western blotting (**Figure 2C**). While this result is consistent with previous work on U20 from HHV-6A (10), and an overall reduction in ULBP1 surface staining observed after HHV-6B infection (9), the lack of a decrease in total ULBP1 protein levels accompanying reduced surface staining suggests that a more complex mechanism is involved beyond simple degradation.

### HHV-6B infection reduces ULBP1 surface staining without decreasing total protein levels

**Figure 3:**
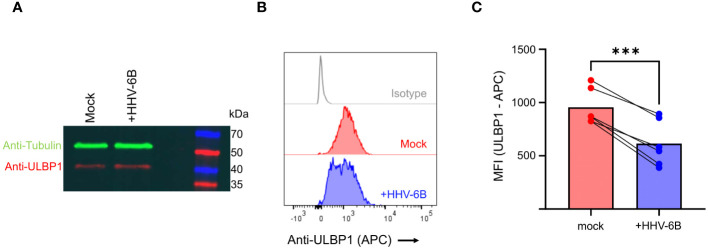
HHV-6B infection decreases surface staining of ULBP1 without decreasing total ULBP1 levels. **(A)** Anti-tubulin (green) and anti-ULBP1 (red) western blot of mock and HHV-6B infected cell. **(B)** ULBP1 and ULBP1/U20 expressing cells were stained with APC conjugated anti-ULBP1 (R&D Systems FAB1380A). Representative flow cytometry data showing the level of anti-ULBP1 mAb binding to ULBP1 expressing cells. **(C)** MFIs were plotted for three independent experiments and the mean ± SD are shown. Statistical analysis is a one-tailed, paired t test, ***p<0.001.

In order to determine whether similar phenomenon are observed in the context of viral infection, we infected SupT1 cells with the Z29 strain of HHV-6B. Infection was confirmed by RT-PCR against immediate early, early, and late genes (**Supplementary Figure S2A**) and efficiency was measured by flow cytometry using an antibody against the viral gB protein (**Supplementary Figure S2B**). First, we performed an anti-ULBP1 western blot, which showed that ULBP1 protein levels do not decrease during infection, and in fact increase by 20% relative to a tubulin control when compared to the mock infected cells (**Figure 3A**). Next, we measured ULBP1 levels by flow cytometry by staining for ULBP1 at the cell surface. In the infected cells, we observed a 37% decrease in ULBP1 staining after infection compared to the mock-infected controls. In this experiment, the infection efficiency was 47.6% (**Supplementary Figure S2B**) These findings confirm that ULBP1 is not degraded during HHV-6B infection and that any surface downregulation of ULBP1 must utilize an alternative mechanism.

### Transduced U20 blocks NKG2D recognition of cell-surface ULBP1

**Figure 4:**
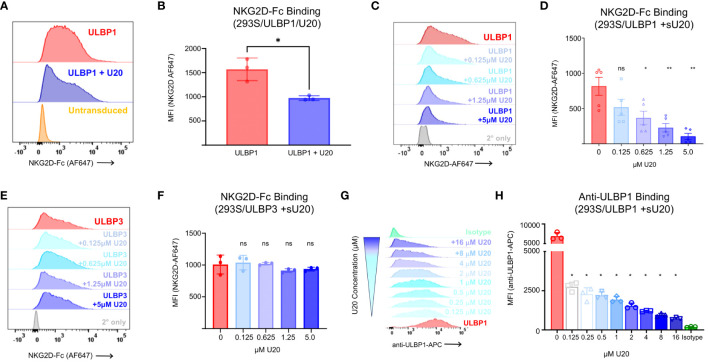
Soluble U20 inhibits binding of NKG2D to cell-surface ULBP1. **(A)** Representative flow cytometry data showing level of NKG2D-Fc binding to HEK293 cells transduced with ULBP1 ± U20. **(B)** MFIs were plotted for three independent NKG2D-Fc binding experiments. The mean ± SD is shown. Statistical analysis for paired t test, *p < 0.05. C. Representative flow cytometry data showing level of NKG2D-Fc binding to HEK293 cells transduced with ULBP1 and pretreated with varying concentrations of sU20. **(D)** MFIs were plotted for five independent NKG2D-Fc binding experiments. The means ± SD are shown. Statistical analysis for repeated measures one-way ANOVA, **p < 0.01, *p < 0.05, ns, not significant. **(E)** Representative flow cytometry data showing level of NKG2D-Fc binding to HEK293 cells transduced with ULBP3 and pretreated with varying concentrations of sU20. **(F)** MFIs were plotted for three independent NKG2D-Fc binding experiments. The means ± SD are shown. Statistical analysis for repeated measures one-way ANOVA, ns, not significant. **(G)** ULBP1 expressing cells were pretreated with various concentrations of soluble U20 before staining with APC conjugated anti-ULBP1 (R&D Systems FAB1380A). Representative flow cytometry data showing the level of anti-ULBP1 mAb binding to ULBP1 expressing cells. The concentration of U20 used for pretreatment is indicated on the right. **(H)** MFIs were plotted for three independent experiments and the mean ± SD are shown. Statistical analysis for repeated measures one-way ANOVA with 0μM U20 set as negative control, *p < 0.05.

To determine if the reduced surface expression of ULBP1 induced by U20 transduction impacted recognition by NGK2D, its activating receptor on NK cells, we stained the transduced cells with soluble NKG2D-Fc. Soluble NKG2D labeling revealed a 38% decrease in NKG2D binding in the presence of U20. A representative experiment is shown in **Figure 4A**, with all replicates quantified in **Figure 4B**. We replicated these experiments in HEK293T cells to rule out any role for fully intact, complex glycans on U20 with similar results (**Supplementary Figure S3**). Thus, transduced U20 can interfere with NKG2D recognition of cell-surface ULBP1 (**Figures 4A, B**) without affecting total protein levels (**Figures 2**, **3**).

### Soluble U20 blocks NKG2D binding to cell-surface ULBP1

With the knowledge that U20 binds directly to ULBP1 and that this interaction decreases NKG2D binding without affecting levels of ULBP1 at the surface or *in toto*, we set out to understand how U20 interferes with NKG2D binding to ULBP1. Recent work on the murine cytomegalovirus (MCMV) m152 immunoevasion protein showed that m152 can block RAE-1γ (a mouse ULBP homologue) from binding to NKG2D, attributed at least in part to m152 masking RAE-1γ at the cell surface (32). To determine if surface masking was a possible mechanism for U20, we pretreated ULBP1-transduced HEK293S GnTI cells for 5 minutes with varying concentrations of purified, soluble U20 (sU20) and then stained with NKG2D-Fc. We observed a concentration-dependent reduction in NKG2D binding as we added increasing amounts of sU20 (**Figures 4C, D**) with 5 μM sU20 decreasing NKG2D binding by almost 90%. Conversely, sU20 exerted no significant effect on NKG2D-Fc binding to ULBP3 expressing cells (**Figures 4E, F**). The finding that sU20 reduces NKG2D binding to ULBP1 is consistent with a surface masking mechanism, but the possibility remained that sU20 binds directly to NKG2D, possibly interfering with its recognition of ULBP1. For example, the cowpox CPXV018 protein has been shown to bind directly to NKG2D, possibly interfering with its recognition of ULBP1 (33). To rule out the possibility that U20 might bind directly to NKG2D, we examined sU20 binding to immobilized NKG2D-Fc by SPR and observed no specific binding of U20 to immobilized NKG2D-Fc up to 16 μM (**Supplementary Figure S1B**). Thus, we conclude that sU20 binds to cell-surface ULBP1 and that this interaction blocks NKG2D binding.

### Soluble U20 competes with a monoclonal antibody for ULBP1 binding

A previous report has suggested that expression of HHV-6A U20 results in degradation of ULBP1 (10). However, these findings relied entirely on flow cytometry-based antibody staining of ULBP1 at the cell surface. We hypothesized that their observation of decreased ULBP1 surface levels in HHV6A U20-expressing cells was due to competition between U20 and the anti-ULBP1 antibody used to detect ULBP1 at the cell surface. To test this hypothesis, we pre-treated ULBP1 expressing cells with increasing concentrations of soluble HHV-6B U20 and then the cells with the same anti-ULBP1 antibody used in the previous study. We observed a dose dependent reduction in antibody binding (**Figure 4G**) and found that as little as 0.125 μM sU20 was sufficient to reduce antibody binding by 60% (**Figure 4H**).

### U20 inhibits NK cell responses to ULBP1-expressing Jurkat T cells

**Figure 5:**
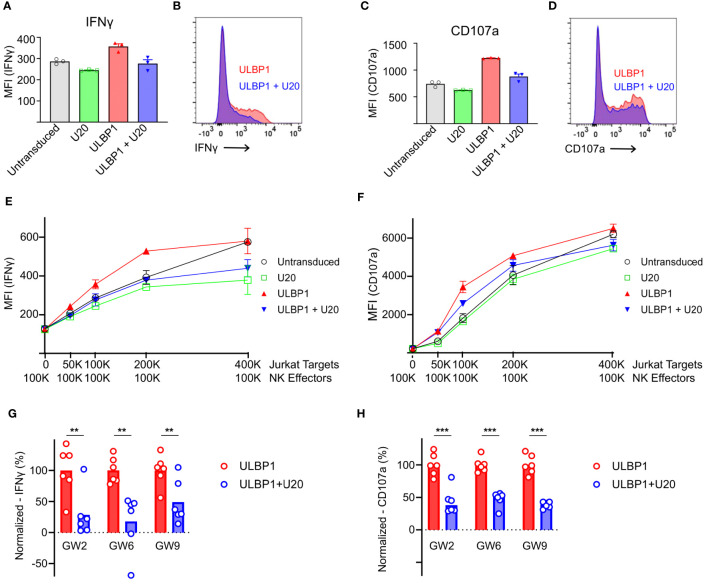
U20 inhibits NK cell responses to ULBP1-expressing Jurkat T cells. **(A)** Representative flow cytometry data comparing IFNγ staining for ULBP1 (red) and ULBP1 + U20 (blue). **(B)** Histogram showing combined triplicates for IFNγ staining of NK cells from donor GW6 performed on a single day. Stimulation with untransduced Jurkat cells is shown in gray, U20 only is shown in green, ULBP1 only is shown in red, and ULBP1+U20 is shown in blue. **(C, D)** As in panels **(A, B)** but showing CD107a staining instead of IFNγ. **(E)** IFNγ levels resulting from stimulation of donor GW6 at various effector to target ratios. The mean ± SD values are shown for triplicate stimulations performed on a single day. **(F)** As in panel **(E)** but for CD107a. **(G)** Normalized percent downregulation of IFNγ levels from Jurkat-stimulated NK cells at a 1:1 E:T ratio. Each donor was tested three times on two separate days. MFIs were normalized by subtracting the signal from the corresponding U20 only wells and dividing by the average ULBP1-only value in each experiment. Statistical analysis is a one-tailed, paired t test, **p < 0.01. **(H)** As in panel **(G)**, but for CD107a, ***p < 0.001.

To measure the effects of U20 on NKG2D-mediated NK cell activity in a cellular model relevant for NK surveillance of HHV-6B infection, we isolated NK cells from healthy donor PBMCs and evaluated their response to Jurkat T cells, using two readouts of NK cell activation: secretion of interferon-gamma (IFNγ), a key NK cell inflammatory cytokine, and surface mobilization of CD107a, a lysosomal protein that traffics to the cell surface accompanying release of cytolytic granules (Gating strategy shown in **Supplementary Figure S4A**). Jurkat cells express several NK activating ligands including ULBP1, ULBP2, and MICA (9, 34–36) and induced NK cells to secrete IFNγ and mobilize CD107a (**Figure 5A**, gray bars). These responses were reduced somewhat by transduction of U20 (**Figure 5C**, green bars). To isolate the effect of U20 transduction on ULBP1-mediated NK activation, we first transduced Jurkat cells with ULBP1, resulting in increased levels of IFNγ secretion and CD107a mobilization (**Figures 5A, C**, red bars). Subsequent transduction of U20 into these cells largely reversed the increased activation induced by transduced ULBP1 (**Figures 5A–D**, compare red and blue bars and FACS profiles). These effects were observed over a range of effector-to-target ratios (**Figures 5E, F**). NKG2D levels vary across the total NK cell population in peripheral blood and between different individuals, but NK populations isolated from several donors all revealed U20-induced reductions in ULBP1-driven NK cell IFNγ secretion and CD107a mobilization (**Figures 5G, H**). The U20-induced reduction in NK activation, expressed as a percentage of the NK activation induced by ULBP1 transduction relative to the level observed for U20 transduction alone, averaged 56–82% for IFNγ secretion and 55–75% for CD107a mobilization across replicate NK samples from three donors (**Figures 5G, H**). Thus, the reduction in ULBP1 surface staining and NKG2D binding observed after U20 expression also impacts functional NK cell responses.

### U20 and ULBP1 form a stable complex

**Figure 6:**
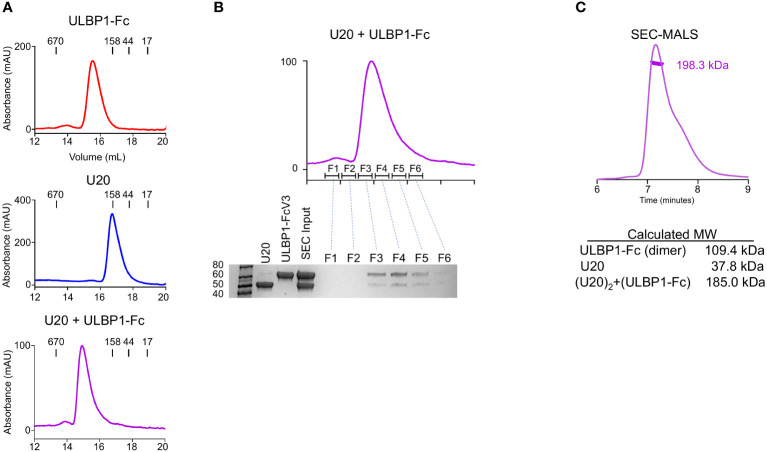
U20 forms a stable complex with ULBP1. **(A)** Size exclusion chromatography traces for ULBP1-Fc, U20, and an equimolar mixture of U20 and ULBP1-Fc. **(B)** Fractions from the SEC run shown in panel **(A)** (right panel) were subjected to SDS-PAGE and stained with Coomassie Blue. **(C)** SEC-MALS analysis of the U20+ULBP1-Fc complex. The measured molecular weight is shown, along with the calculated molecular weight of U20, ULBP1, and a complex composed of two molecules of U20 and one molecule of ULBP1-Fc.

We next set out to understand the biochemical basis for the interaction of U20 with ULBP1 and how U20 interferes with NGK2D recognition. We began by characterizing the U20-ULBP1 complex using size-exclusion chromatography. In this experiment we used soluble recombinant U20 produced in HEK293S GnTI cells carrying the full complement of N-linked glycans (37). Analysis by mass spectrometry of U20 treated with various glycosidases confirmed the presence of nine N-linked glycosylation sites (**Supplementary Figure S1C**, **Supplementary Table S1**). U20 and ULBP1-Fc were combined in an equimolar ratio, incubated at room temperature for 1 hour, and passed over a Superose 6 Increase column along with the individual U20 and ULBP1-Fc samples in separate runs. The U20 and ULBP1-Fc mixture produces a clean peak that elutes earlier than either of the individual components, indicating that a complex is formed (**Figure 6A**). To confirm this, we subjected each fraction of the complex peak to reducing SDS-PAGE. The complex peak (fractions F3-F5) is clearly a mixture of both U20 and ULBP1-Fc (**Figure 6B**).

To investigate the stoichiometry of the complex we used multi-angle light scattering coupled to size exclusion chromatography (SEC-MALS) to estimate an absolute molecular mass (**Figure 6C**). The 9 N-linked glycosylation sites of U20 complicate precise molecular weight calculations because of heterogeneous glycosylation. The HEK293S GnTI cells that we used for protein expression lack functional N-acetylglucosaminyltransferase I, resulting in high-mannose glycans that allow for facile deglycosylation via the endoglycosidase H (EndoH). This treatment leaves a single N-acetylglucosamine (GlcNAc) residue of 221 Da at each glycosylated asparagine. SEC-MALS analysis of the deglycosylated U20/ULBP1-Fc complex yielded a molecular weight of 198.3 kDa (**Figure 6C**). The calculated molecular weight of (ULBP1)_2_-Fc with intact high mannose glycans is 109.4 kDa and that of soluble EndoH-treated U20 is 37.8 kDa. The theoretical weight of a complex with each ULBP1 arm engaging one U20 would be 185.0 kDa (**Figure 6C**), consistent with the observed apparent molecular weight and suggesting that U20 and ULBP1 form a 1:1 complex.

### Soluble U20 forms a dimer in solution

**Figure 7:**
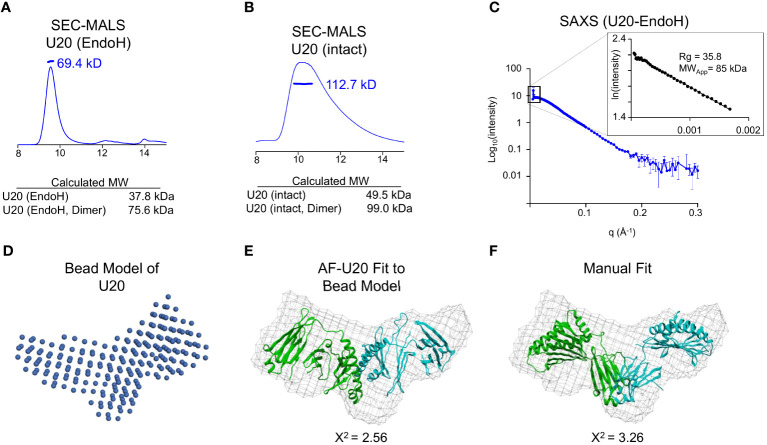
Soluble U20 exists as a homodimer. **(A)** SEC-MALS trace of glyco-intact treated U20. The measured molecular weight is shown, as well as the calculated molecular weight of the monomeric and dimeric forms of U20 with low mannose glycans. **(B)** SEC-MALS trace of EndoH treated U20. The measured molecular weight is shown, as well as the calculated molecular weights of the monomeric and dimeric forms of U20. **(C)** SEC-SAXS scattering data for U20 with corresponding Guinier plot shown in the inset. The radius of gyration and molecular weight determined by the Porod volume method are shown. **(D)** Bead model of the U20 dimer generated by DAMMIF. **(E)** U20 dimer model generated by AlphaFold Multimer fit into a volume generated using the U20 bead model. The chi-square value output from Crysol describing the goodness of fit of the model to the experimental data is shown. **(F)** Two U20 monomers from the AlphaFold database were fit manually into a volume generated using the U20 bead model. The chi-square values output from Crysol describing the goodness of fit of the models to the experimental data are shown.

In the size-exclusion chromatography experiment shown in **Figure 5A**, glycosylated U20 elutes much earlier than would be expected for a protein of ~49.5 kDa. Thus, we were prompted to investigate whether soluble U20 exists as an oligomer in solution. We used SEC-MALS to determine the molecular mass of U20 in solution and test this possibility. As described above, we treated U20 expressed in GnTI cells with EndoH to reduce uncertainty due to heterogenous glycosylation. EndoH-treated U20 measured 69.4 kDa (**Figure 7A**), consistent with the calculated molecular weight for a deglycosylated U20 dimer (~76.0 kDa). To evaluate whether glycans played a role in oligomer formation, we used SEC-MALS to measure the molecular weight of U20 with intact high mannose glycans (**Figure 7B**). The resultant value of 112.7 kDa is consistent with the calculated molecular weight of U20 with 9 high mannose glycans of ~100.0 kDa, suggesting that glycosylated U20 also forms a dimer in solution.

To gain further insight into the structure of the U20 homodimer, we utilized small angle X-ray scattering (SAXS). SAXS provides information on the overall size of the molecular species in solution, like SEC and SEC-MALS, but it also allows for low resolution modeling and comparison with existing structures. The LiX beamline at the National Synchrotron Light source NSLS-2 provides SAXS analysis coupled to size exclusion chromatography (SEC-SAXS), so that individual species in mixtures can be isolated and characterized, with wide angle X-ray scattering (WAXS) data also collected to allow for better subtraction of signal from the sample buffer. We collected a SEC-SAXS/WAXS dataset for EndoH-treated U20 that includes data extending to q=3 Å^-1^, although we truncated the background-subtracted scattering curve to a resolution of q=0.3 Å^-1^ for subsequent analysis (**Figure 7C**). The linear Guinier plot of the low-resolution region (inset to **Figure 6C**) reveals a single species with radius of gyration 35.8 Å, corresponding to a globular protein of approximately 75.3 kDa using the Porod volume determination method (38, 39). This confirms formation of a dimer, as suggested by the SEC-MALS data.

The X-ray scattering profile was used to generate a bead model, using the program DAMMIF (24, 25). We imposed P2 symmetry since our data indicated that U20 exists as a dimer in solution, resulting in the bead model representation shown in **Figure 7D**. Twenty independent DAMMIF bead models were averaged using DAMAVER (26) to obtain a consensus molecular envelope. We compared the resulting averaged bead model with potential models of a U20 dimer. Since no direct structural information is available for HHV-6B U20 or any homologous protein, we began with models generated by various machine-learning structure prediction algorithms (**Supplementary Figure S5**). We generated a model for a U20 dimer using two different methods. First, we utilized AlphaFold2 Multimer which can simultaneously fold multiple copies of a target sequence in an oligomeric configuration (40). This model predicted the U20 MHC-like domains apposed in an unconventional manner and fit reasonably well into the consensus molecular envelope (X^2^ = 2.56), represented as a mesh in **Figure 7E**.

We also considered models for monomeric U20 generated by AlphaFold2, RoseTTAFold, and ESMFold (**Supplementary Figure S5**) (18–20). Pairs of these models were manually fit to the consensus envelope. As previously reported for RoseTTAFold and an earlier iteration of AlphaFold, machine-learning models for U20 generally predict an MHC-like platform domain linked to an immunoglobulin-like domain, consistent with earlier structural modelling efforts, but with greatly varying interdomain angles (**Supplementary Figure S5**) (3, 41). To accommodate this uncertainty, we fit isolated MHC-like platform domains and Ig-like domains to the consensus envelope, considering constraints on interdomain distance imposed by the primary structure. The best fitting models suggested apposed Ig-like domains.

To assess the fitness of all potential dimer models, we generated theoretical scattering profiles using Crysol (27) and compared them to the actual scattering data we collected for U20. The dimer model generated by AlphaFold2 Multimer fit well, with a chi-square value of 2.56 (**Figure 7E**). Our best manually oriented model had a chi-square value of 3.26 (**Figure 7F**), with the Ig-like domains apposed. Thus, none of the models fit the consensus envelope within its expected uncertainty (i.e. with chi-square ~1). Given the uncertainties in bead model generation and computational structure prediction, it was not clear which of models shown in **Figures 7E, F** would be preferred, and other arrangements (including less symmetrical ones) might also fit within the same envelope. However, regardless of the exact configuration, it seems clear from the size and shape of the consensus envelope that the only possible composition of the solution oligomer is two molecules of U20.

### Stoichiometry of the U20-ULBP1 heterodimer

We next revisited the issue of stoichiometry of the U20:ULBP1 complex given the propensity of U20 itself to dimerize in solution. As described above, we observed that U20 and ULBP1-Fc form a complex consisting of two molecules of U20 and one molecule of (ULBP1)_2-_Fc. Molecular interpretation of this result is complicated because of U20’s dimeric nature. It is unclear whether the U20 dimer binds two molecules of ULBP1, or if the U20 dimer dissociates to form 1:1 U20-ULBP1 heterodimers, or if a single U20 dimer binds to one of the one ULBP1 arms but the arrangement of the ULBP1-Fc molecule prohibits binding of a second U20 dimer to the adjacent ULBP1 arm. To address more clearly the question of U20-ULBP1 binding stoichiometry, we characterized the interaction of U20 with soluble monomeric ULBP1 that contains a 6xHis tag instead of the Fc domain.

**Figure 8:**
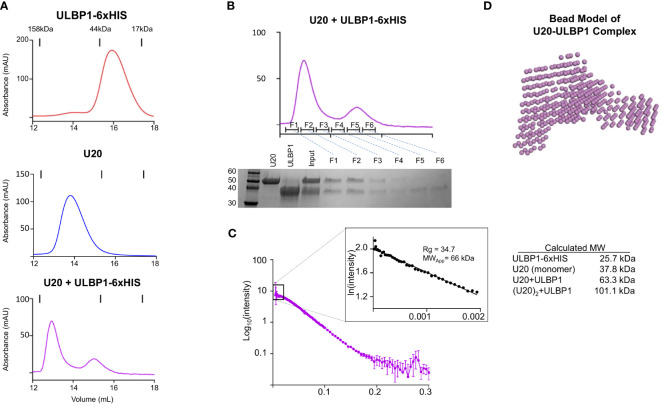
U20 and ULBP1 form a 1:1 heterodimer. **(A)** SEC traces of ULBP1–6H, U20, and an equimolar mixture of U20 and ULBP1–6H. All proteins were deglycosylated with EndoH. **(B)** Fractions from the SEC run shown in **(A)** (bottom panel) were subjected to SDS-PAGE and stained with Coomassie Blue. **(C)** SEC-SAXS scattering data for U20/ULBP1–6H with corresponding Guinier plot shown in the inset. The measured molecular weight is shown along with a table containing the weights of the complex components and the theoretical weight of various complex stoichiometries.

As before, for the Fc complex, we combined equimolar amounts of ULBP1–6H and U20, incubated them for 1 hour at room temperature, and subjected them to size exclusion chromatography (**Figure 8A**). A shift in elution volume is observed relative to the individual components. Fractions from the SEC run of the complex were analyzed by SDS-PAGE, showing that the new peak is composed of both U20 and ULBP1 (**Figure 8B**). In order to definitively answer the question of stoichiometry, we once again turned to SEC-SAXS. We would expect a 1:1 complex to have a molecular weight of 63.5 kDa (**Figure 8C**). Analysis of the scattering data indicated a single species with a radius of gyration of 34.7 Å, corresponding to a globular protein of approximately 66.0 kDa (**Figure 8C**). This result is consistent with a complex in which the U20 dimer breaks apart to form 1:1 heterodimers each with a single molecule of ULBP1.

### Modeling the U20-ULBP1 Complex

We used the SEC-SAXS data to develop a molecular model of the U20+ULBP1–6H complex. As described previously, we generated a bead model using DAMMIF and DAMAVER (**Figure 8A**) and began producing mockups of potential complex arrangements. Initially we tried to use AlphaFold2 Multimer to model a U20/ULBP1 heterodimer. However, the AlphaFold2 Multimer algorithm produced only models in which the two proteins were very tightly packed together in a globular shape, while the bead model appeared to be more elongated (compare **Supplementary Figure S5D**, **Figure 8D**). When we assessed the fit of these AlphaFold2 Multimer models to the experimental data using Crysol, the theoretical scattering curves were inconsistent with the experimental data and produced chi-square values ranging from 14.8 to 20.8 (**Supplementary Figure S5D** inset).

We next tried manual fitting using the U20 models that we had already compiled (**Supplementary Figure S5**) and a model of ULBP1 from the AlphaFold database (42), which very closely matched previously determined crystal structures of the homologous ULBP3 and ULBP6 proteins. We hypothesized that ULBP1 would interact with NKG2D similarly to the previously characterized structures of NKG2D in complex with ULBP3, ULBP6, and MICA (43–45), with the saddle shaped NKG2D dimer interacting with the α1 and α2 helices of each NKG2D ligand. The fact that U20 blocks NKG2D from binding to ULBP1 significantly constrains the number of possible orientations, since a portion of U20 must cover some part of the NKG2D binding site on top of the ULBP1 α1 and α2 helices. Additionally, oriented ULBP1 in the bead model using the shape of the consensus envelope and the long C-terminal tail present on the ULBP1 construct, which further constrains the position of U20 binding to the opposite side of ULBP1. Finally, we considered U20’s glycans, ensuring that all nine glycosylated asparagine residues were positioned in such a way as to not overlap with ULBP1, since binding was not affected by removal of the glycans. None of the intact unmodified U20 models fit well into the envelope, with the best fit model having X^2^ = 14.8. Therefore, we separated the platform and Ig domains as described previously and sampled a range of interdomain angles. Unexpectedly, the best fits were obtained when the C-terminal tail of U20, which contains the 6xHIS tag, projected out and away from the rest of the molecule. We tested several models with the tail of U20 in various orientations, examples of which are shown in **Supplementary Figures S6A** and **B**.

Additionally, we tested similar movements of the tail of ULBP1. A 90-degree pivot had a small effect on the chi-square value (**Supplementary Figure S6C**) but the outright removal of ULBP1’s tail resulted in a worse fit than what was observed in the case of U20 (**Supplementary Figure S5**), indicating a more significant effect on the fit to the experimental data. This suggests that the tail of ULBP1 could be less flexible than U20’s and that it adopts a smaller number of possible conformations, each of which contributes more to the scattering profile than the tail of U20.

**Figure 9:**
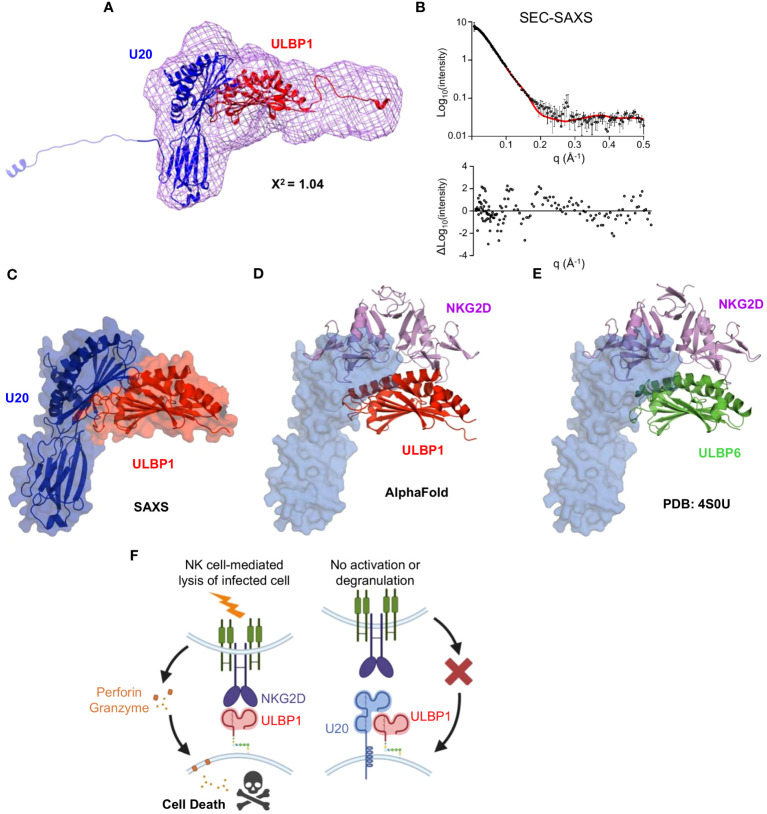
Modeling the U20/ULBP1 complex. **(A)** The platform domain of ESMFold U20 (blue, top), Ig domain of AlphaFold U20 (blue, bottom), and AlphaFold model of ULBP1–6H (red) were fit manually into a volume generated using the U20 bead model. This configuration represents the best fit of the 25 models tested. **(B)** The chi-square value output from Crysol describing the goodness of fit of the model to the experimental data is shown, as is the theoretical scattering curve and fit residuals. **(C)** The U20/ULBP1 model shown in **Figure 8A** was rotated to better visualize the location of the NKG2D and the C-terminal tails were removed. **(D)** Model of ULBP1 (red) bound to the NKG2D dimer (purple) generated by AlphaFold. U20 (in blue) was placed at its predicted binding site on ULBP1. **(E)** The previously solved crystal structure of ULBP6 (green) bound to the NKG2D dimer (purple) (44). U20 (in blue) was positioned according to its predicted binding site on ULBP1. **(F)** Model for U20 evasion of NK recognition by binding ULBP1 and inhibition of NGK2D by a surface masking mechanism.

Finally, when we combined the U20 platform domain from the ESMFold model combined with the Ig domain from the AlphaFold3 database prediction for U20 we achieved the best fit by far (**Figure 9A**). Comparison of predicted and observed scattering curves using Crysol fitting yielded a chi-square value of 1.0, with a largely random distribution of residuals (**Figure 9B**). In this model, the underside of the platform domain of U20 makes contact with the α1 and α2 helices of ULBP1 while the Ig domain of U20 projects underneath and slightly away from ULBP1 (**Figure 9C**). Although no structure is available for the ULBP1/NKG2D interface, examination of an AlphaFold model of the ULBP1/NKG2D complex (**Figure 9D**) and a crystal structure of the homologous ULBP6/NKG2D complex (**Figure 9E**) reveals that U20 binding to ULBP1 would block a significant portion of the NKG2D binding site. When taken together, our results suggest the mechanism depicted in **Figure 9F**, wherein U20 binds with high affinity to ULBP1, blocking NKG2D binding to ULBP1 and preventing NK cells from becoming activated.

## Discussion

In this work, we describe the role of U20, a viral immunoevasin expressed by HHV-6B, in inhibiting NK cell activation by masking ULBP1 from detection by NKG2D. We first used purified recombinant proteins to show that U20 binds directly to ULBP1 with sub-micromolar affinity. In cellular assays, we demonstrated that U20 inhibits NKG2D binding to ULBP1 but does not decrease the total amount of ULBP1. Then, using soluble U20, we showed that U20 could inhibit NKG2D binding to ULBP1 in a concentration-dependent manner. Finally, we showed that U20 interferes with ULBP1-stimulated activation of primary NK cells. These findings strongly suggest that U20 utilizes a surface masking mechanism to block ULBP1 from recognition by NKG2D. Finally, by combining small-angle X-ray scattering (SAXS) with computational structural predictions, we generated a model of the U20-ULBP1 complex consistent with the experimental data.

A surface masking mechanism was unexpected, as a previous report suggested that HHV-6A U20 decreases surface levels of ULBP1 (10). Other herpesvirus MHC-like immunoevasins function by facilitating sequestration or degradation of their targets, including HHV-7 U21 and HCMV US2, which downregulate MHC-I, and HCMV UL16, which downregulates NKG2D ligands including ULBP1 (3). The binding of U20 to ULBP1 competes with anti-ULBP antibody binding (**Figures 4G, H**), possibly explaining previous reports of reduced surface staining. Moreover, ULBP1 downregulation by HHV-6A U20 was not affected by proteasomal or lysosomal inhibitors, ruling out a degradation-based mechanism (10). We show that the binding affinity of U20 for ULBP1 is approximately 4-fold stronger than that of NKG2D for ULBP1 [**Figure 1** and (30)], suggesting that U20 could compete effectively with NKG2D for binding at the cell surface. ULBP1 has a relatively short cell-surface lifetime before being internalized or shed from the cell membrane (46), making it more susceptible to a surface-masking mechanism.

Although sequestration or degradation is a common herpesvirus immunoevasion strategy, there is precedent for viral surface-masking. The m152 glycoprotein expressed by mouse cytomegalovirus (MCMV) had been thought to function exclusively by causing intracellular retention of the mouse ULBP homolog RAE-1 (47). However, a recent study showed that m152 can utilize a surface masking mechanism in addition to this retention mechanism if the conventional pathway becomes saturated (32). In the case of U20, surface masking of ULBP1 appears to be the primary mechanism by which U20 inhibits NK cell recognition of ULBP1 (**Figures 2**–**4**); however, we cannot rule out the possibility that there are other means by which U20 inhibits ULBP1.

We used SEC-SAXS data and the U20-ULBP1-NKG2D binding results to develop a structural model of the U20-ULBP1 complex. The relatively high level of uncertainty in the U20 model and the low resolution of the diffraction data make residue-level conclusions uncertain. However, we believe that this model represents a starting point for further investigation. That U20 forms a dimer in solution raises some interesting questions: previous work modeling the structure of the U20 extracellular domain suggested that it could contain an MHC-like fold, but many regions were not modeled confidently, and the relative orientation of the domains was uncertain (3). Whether these regions are unstructured or just difficult to model by AlphaFold’s co-evolution distance matrix approach is unclear (40). If U20 does have intrinsically disordered regions that require a binding partner for stability, this could explain some of the difficulties of predicting a fully folded structure. It remains to be seen whether full-length membrane-bound U20 forms a dimer as observed for the soluble extracellular domain, and if so, whether the dimer has any physiological role. Finally, the model of ULBP1 (**Supplementary Figure S5**) also contains poorly-modeled regions, particularly two loops underneath the α1 helix, and it is possible that these could move to make contact with flexible regions of U20.

To successfully establish a foothold within their host, herpesviruses must evade the immune response. HHV-6 downregulation of MHC-I was first reported in 2001 (48), and observations of NK cell responses driven by the loss of MHC-I even earlier (49). NK cells are known to respond to HHV-6B infection (50, 51), and HHV-6B must have a means to control their activity. How does HHV-6B inhibit CD8 T cell responses without being eliminated by NK cells? Other herpesviruses, including HCMV (52) and VZV (53), inhibit NK cell responses by interfering with NKG2D-mediated recognition of the MIC and ULBP families of stress ligands expressed on infected cells. These NKG2D ligands would usually alert NK cells to the presence of the virus, allowing them to kill infected cells before the virus can spread. HHV-6B has been reported to downregulate three NKG2D ligands (ULBP1, ULBP3, and MICB), but the mechanism has remained unknown (9). Here, we show that HHV-6B U20 binds to ULBP1, blocking NKG2D interaction as well as surface staining. However, U20 does not exhibit detectable binding to ULBP3 and MICB (**Figure 1**), and how HHV-6B downregulates these ligands remains unknown. Thus, our finding that U20 inhibits NK cell activation by blocking NKG2D-mediated recognition of ULBP1 represents just a first step in understanding how HHV-6B modulates NK cell responses to facilitate lifelong infection.

## Limitations of this study

Although we have demonstrated that U20 binding can mask ULBP1 from recognition by NKG2D, it is possible that U20 could in addition utilize other mechanisms to interfere with NKG2D recognition of ULBP1. While previous studies have shown that U20 does not actively promote the degradation of ULBP1 (10), it is possible that it could promote intracellular retention of ULBP1. Future studies will be required to determine whether surface masking is the only mechanism at play. Finally, while we saw a significant U20-mediated effect on ULBP1-driven NK cell activation, there was some variability between donors. Further studies will be required to understand the implications of this heterogeneity and what it means for HHV-6B in the context of an actual infection.

## Data availability statement

The mass spectrometry data used for identifying N-linked glycosylation sites has been deposited with the ProteomeXchange Consortium via the MassIVE repository with the dataset identifier MSV000094990. All other original contributions presented in the study are included in the article/**Supplementary Material**. Further inquiries can be directed to the corresponding author.

## Ethics statement

The studies involving humans were approved by UMass Chan Medical School Institutional Review Board. The studies were conducted in accordance with the local legislation and institutional requirements.

## Author contributions

GW: Conceptualization, Data curation, Formal analysis, Investigation, Methodology, Project administration, Writing – original draft, Writing – review & editing. CS: Conceptualization, Formal analysis, Investigation, Methodology, Writing – review & editing. AB-A: Writing – review & editing, Methodology, Investigation, Formal analysis. KC: Methodology, Resources, Writing – review & editing. AH: Conceptualization, Methodology, Resources, Writing – review & editing. LS: Conceptualization, Formal analysis, Resources, Writing – original draft, Writing – review & editing.
