# Supplementary material for: The HHV-6B U20 glycoprotein binds ULBP1, masking it from recognition by NKG2D and interfering with natural killer cell activation
Source: Front Immunol. 2024 Jun 17;15:1363156. doi: 10.3389/fimmu.2024.1363156 (PMC11215065; doi:10.3389/fimmu.2024.1363156)
Supplement: Supplementary file 1 [file DataSheet_1.pdf]

Supplementary Material

The HHV-6B U20 glycoprotein binds ULBP1, masking it from recognition by NKG2D and interfering with natural killer cell activation

Grant C. Weaver, Christine L Schneider, Aniuska Becerra-Artiles, Kiera L. Clayton, Amy W. Hudson, and Lawrence J. Stern*

*** Correspondence:** Lawrence Stern: lawrence.stern@umassmed.edu

Contents of Supplementary Material

Supplementary Methods

Supplementary Figure 1: Validation of recombinant, soluble ULBP1 and U20

Supplementary Figure 2: HHV-6B infection of SupT1 cells

Supplementary Figure 3: Cellular U20 inhibits binding of NKG2D to cell-surface ULBP1 in cells with fully intact glycosylation

Supplementary Figure 4: Gating strategy for NK recognition assay

Supplementary Figure 5: Structural models used in this study.

Supplementary Figure 6: Fitting the C-termini of U20 and ULBP1 into SEC-SAXS-derived models

Table S1: U20 N-linked glycosylation sites

Table S2: Staining panel for NK recognition assays

Table S3: RT-PCR Primers

Supplementary References

Supplementary Methods

HHV-6B infection

HHV-6B infected cells were collected by centrifugation at the indicated timepoints, washed with PBS, and total RNA was extracted using the RNeasy kit (QIAGEN Inc., Valencia, CA), following manufacturer's instructions. The procedure included a DNase-I digestion step, to digest any free viral DNA present in the preparation. First strand DNA synthesis was performed using RevertAid First Strand cDNA Synthesis kit (Thermo Scientific Inc.), starting from ~1µg total RNA. RT-PCR was performed using Dream Taq Green PCR Master mix (Thermo Scientific, Inc.) and primers for three viral transcripts (U86, U20, and U12) as well as beta-actin as a reference gene. All primer sequences are included in Table S2 and some are used as previously described [1].

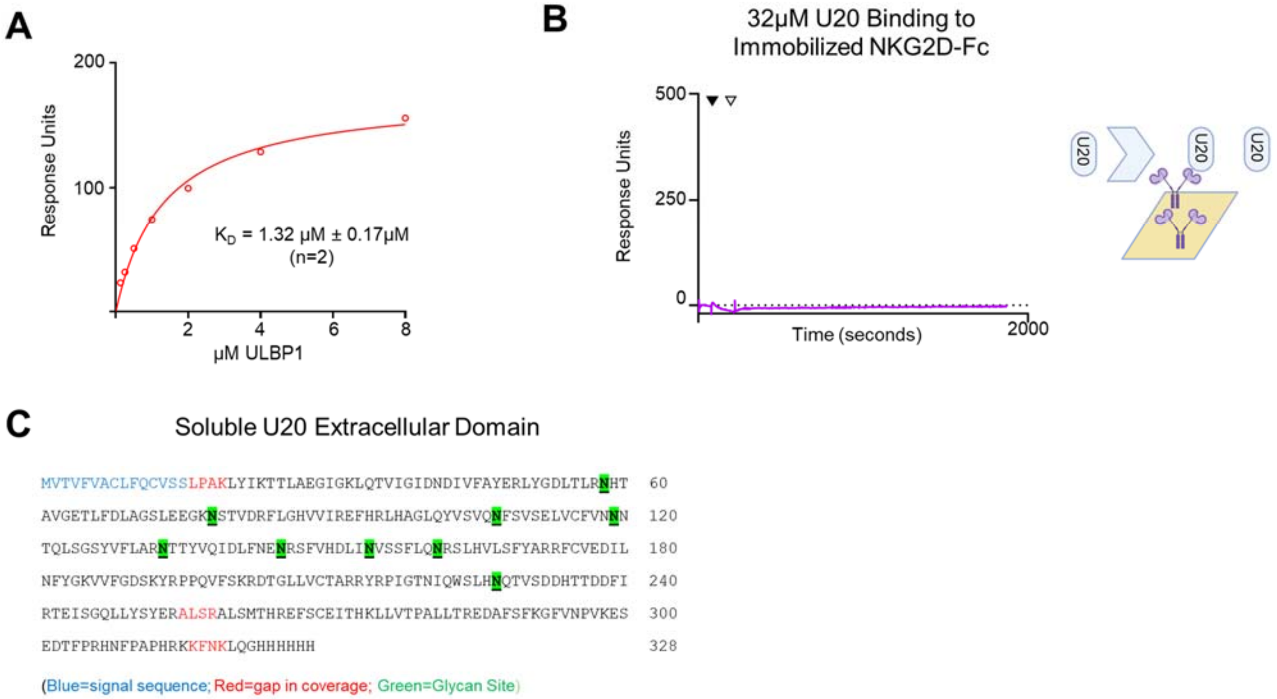

Supplementary Figure 1. Validation of recombinant, soluble ULBP1 and U20. A. Validation of in-house produced ULBP1. ULBP1-Fc was coupled to a neutravidin chip and NKG2D-Fc was injected in twofold dilutions from 0.125 µM to 8 µM. Representative equilibrium binding curve fit. K_Ds were calculated by nonlinear curve fitting and the mean K_D and standard deviation are shown. **B**. SPR analysis of U20 binding to NKG2D. An Fc fusion of NKG2D was coupled to a neutravidin chip at and sU20 was injected at a concentration of 32µM on a Biacore 3000 instrument. **C**. Soluble U20 extracellular domain was subjected to mass spectrometry to identify all of its N-linked glycan sites. The signal sequence is shown in gray, confirmed glycan sites are highlighted in green, and gaps in coverage are indicated in red.

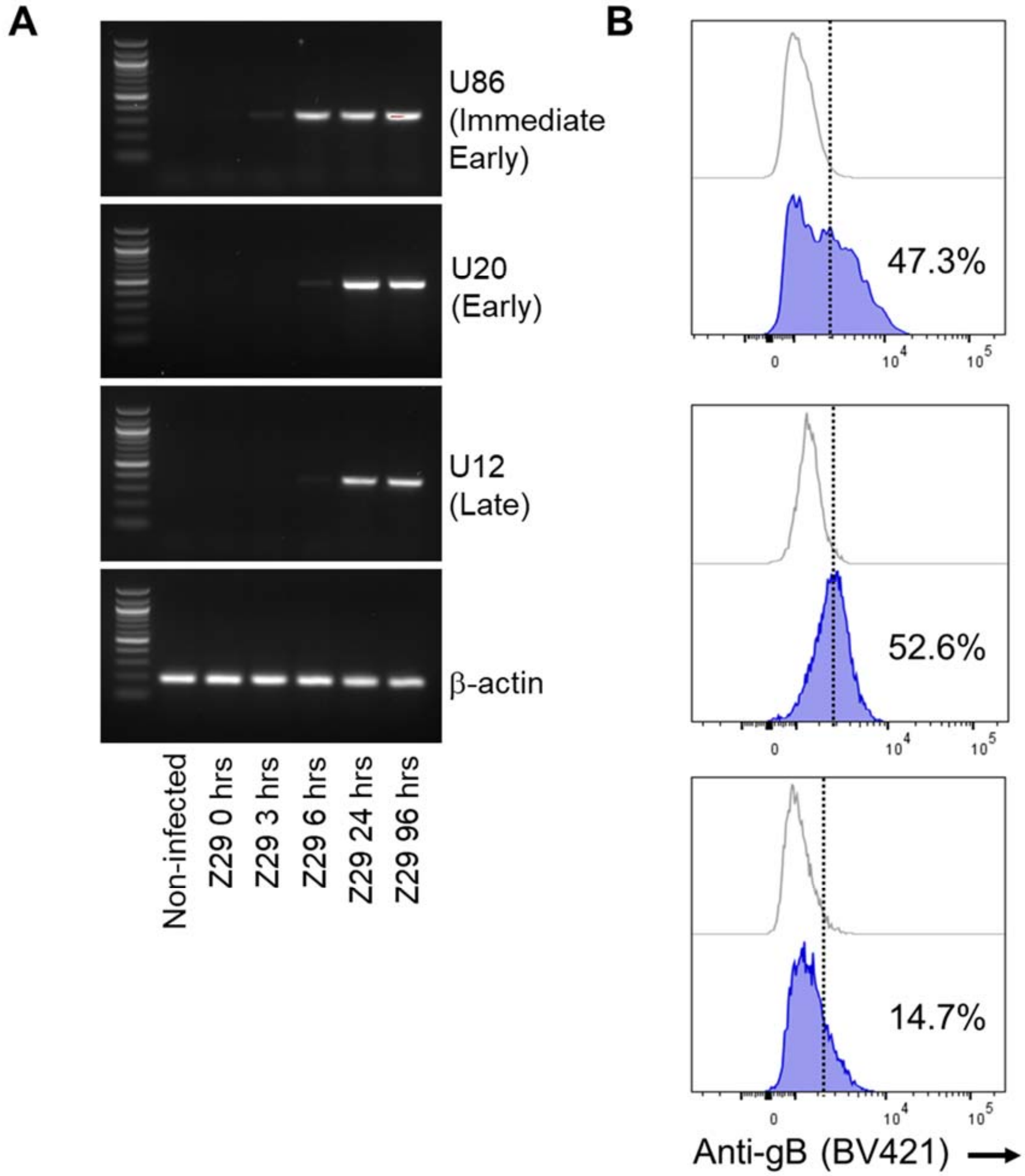

Supplementary Figure 2. HHV-6B infection of SupT1 cells. A. RT-PCR of HHV-6B infected cells. Infected cells were harvested at the indicated timepoints and subjected to RT-PCR to verify expression of the indicated viral genes. B-actin was used as a control. **B.** Verifying viral infection by flow cytometry. Infected cells were stained for the gB glycoprotein at 72 hours post-infection, concurrently with the ULBP1 staining shown in Fig. 3. Each histogram shown represents an independent infection.

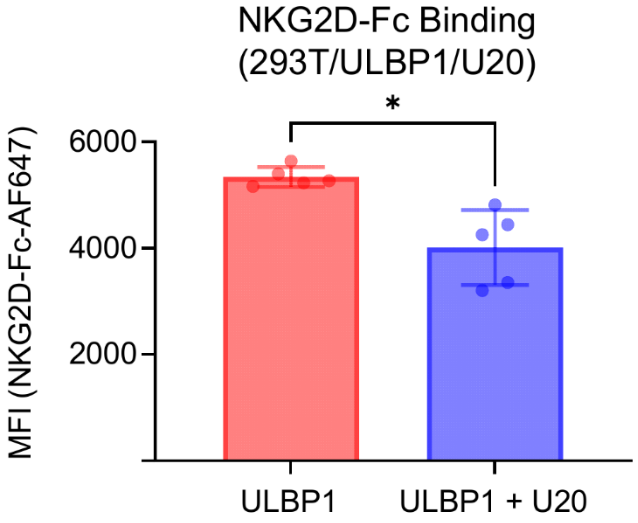

Supplementary Figure 3. Cellular U20 inhibits binding of NKG2D to cell-surface ULBP1 in cells with fully intact glycosylation. A. MFIs were plotted for three independent NKG2D-Fc binding experiments. The means ±SD are shown. Statistical analysis for paired t test, $*p < 0.05$.

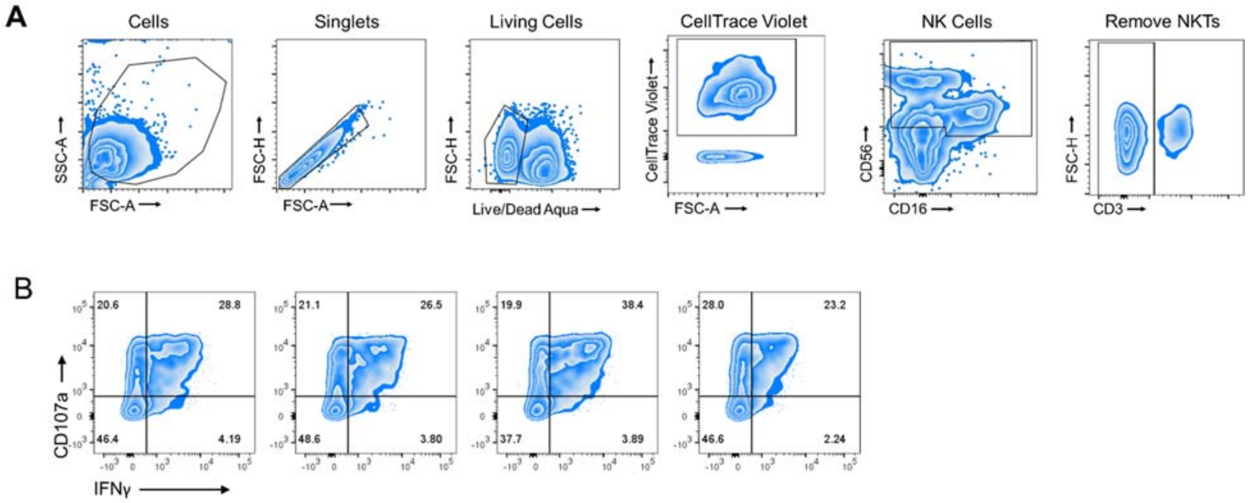

Supplementary Figure 4. Gating strategy for NK recognition assay. A. Gating strategy for NK recognition assay from one representative donor. **B.** Frequency of activated cells as measured by CD107a (y-axis) and interferon-gamma (x-axis) in one representative donor.

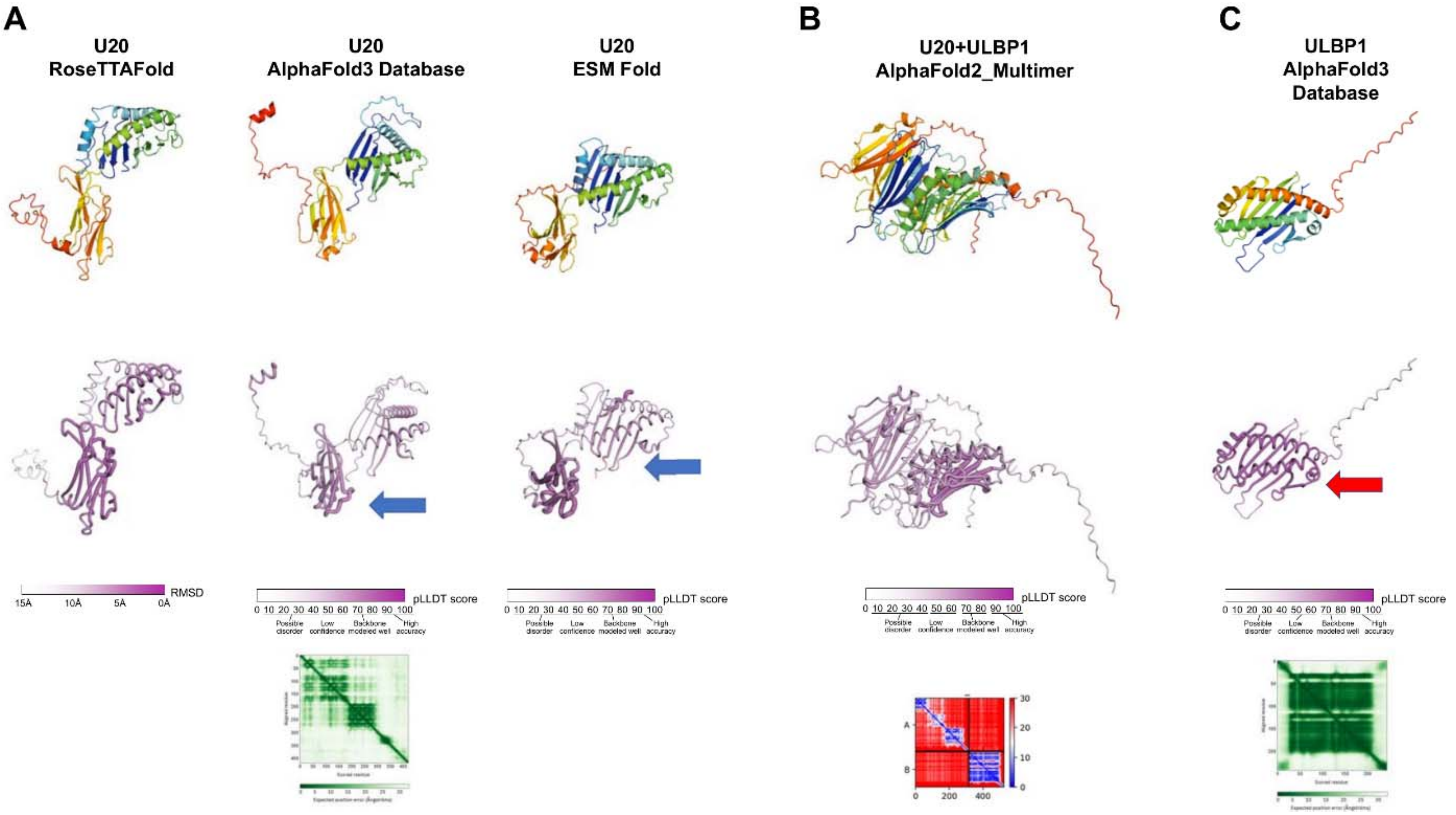

Supplementary Figure 5. Structural models used in this study. The sequences used for prediction were taken from our expression constructs, processed through SignalP to identify signal sequences [2], and truncated to reflect what was actually expressed. The upper are show ribbon diagrams colored by linear protein sequence; bottom panels show tube diagrams colored by confidence score. Scale bars representing the confidence intervals are shown below each model. Arrows indicate domains used in SAXS-based structural studies. **A.** Models of U20 used in this work. **B**. AlphaFold2 Multimer model of a U20/ULBP1 complex generated for this work. **C**. Model of ULBP1 used in this work.

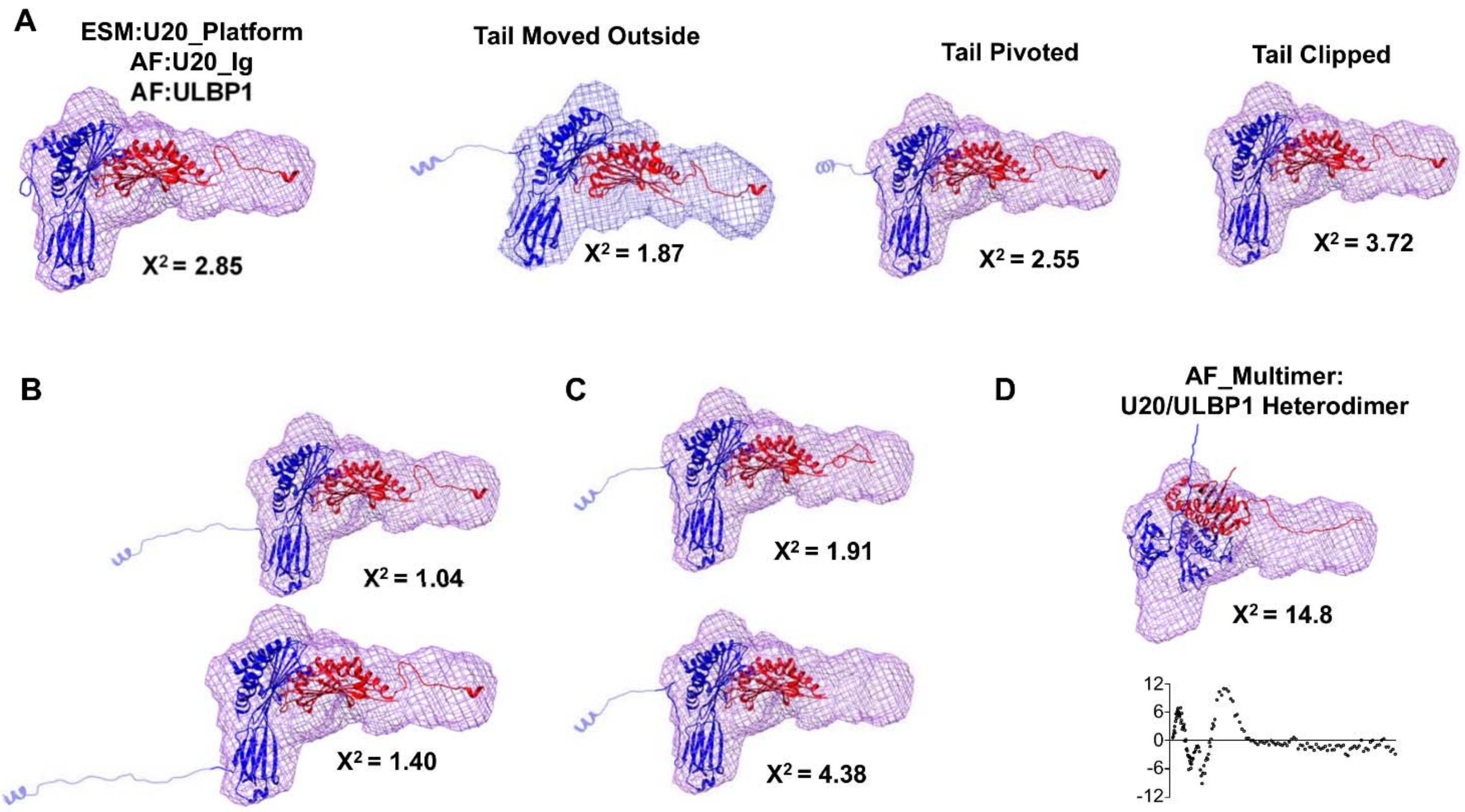

Supplementary Figure 6. Fitting the C-terminal tails of U20 and ULBP1 into SEC-SAXS-derived models. A. Various manipulations of the U20 C-terminal tail were tested for their effects on the goodness of fit: moving part of the tail outside of the envelope, a 90-degree pivot, and outright deletion. **B.** Additional tail bend locations were tested for their effect on goodness of fit**. C.** Various manipulations of the ULBP1 C-terminal tail were tested for their effects on the goodness of fit: a 90-degree pivot (Top) and outright deletion (Bottom). **D**. AlphaFold Multimer was used to generate various models of the U20/ULBP1 complex. The best model resulting from these predictions was tested for goodness of fit with the experimental data.

Peptide	Glycosidase	Protease(s) Used	Modification(s)	Position	Retention Time	Charge	Mass	Intensity
**N**HTAVGETLFDLAGSLEEGK	EndoH	Trypsin	HexNAc	N45	3230	2	2290.1	2.51E+09
**N**HTAVGETLFDLAGSLEEGK**N**STVDR	EndoH	Trypsin	HexNAc, HexNAc	N45/N65	2930	4	3165.5	3.52E+09
LHAGLQYVSVQ**N**FSVSE	PNGaseF	GluC/ Trypsin	Asn>Asp	N94	3070	3	1877.91	2.26E+08
LVCFVN**N**NTQLSGSYVFLAR	EndoH	GluC /Trypsin	HexNAc	N106	3040	2	2504.23	3.04E+10
**N**TTYVQIDLFNE**N**R	EndoH	Trypsin	HexNAc, HexNAc	N120/N132	1067	2	2131.99	3.32E+08
SFVHDLI**N**VSSFLQ**N**R	EndoH	Trypsin	HexNAc, HexNAc	N141/N148	3090	3	2281.13	1.61E+10
RYRPIGTNIQWSLH**N**QTVSDDHTTDDFIR	PNGaseF	Trypsin	Asn>Asp	N214	2340	5	3485.67	4.68E+10

Table S1: Selected peptides derived from U20 extracellular domain to identify N-linked glycosylation sites.

Table S2: Staining panel for NK recognition assays

Antibody	Company	Part Number	Species	Volume per Test
NKG2D-PE/Cy7	Biolegend	320812	mouse	5μl
CD16-BV711	Biolegend	302044	mouse	2μl
CD56-BV650	Biolegend	362532	mouse	2μl
CD3-PerCP/Cy5.5	Biolegend	375109	mouse	5μl
LIVE/DEAD Aqua	ThermoFisher	L34966	NA	4μl per mL of staining solution
Interferon-gamma-APC	Biolegend	502516	mouse	2μl

Primer	Direction	Primer Sequence
U12	Forward	5'-CCTCAATGTGCGCGATTCC-3'
	Reverse	5'-GCAATATCTCTGCACGGGCT-3'
U20	Forward	5'-TGCTTACGAGAGGCTTTATGG-3'
	Reverse	5'-ACTTGCGGAGGACGATATTTAG-3'
U86	Forward	5'-AGCGCACCCGGGATGATGATTCA-3'
	Reverse	5'-TGATGGCTCCGGTGTTAGAGCATCCA-3'
β-actin	Forward	5'-ATCCTCACCCTGAAGTACCCCA-3'
	Reverse	5'-GAAGGTCTCAAACATGATCTGGGT-3'

Table S3: RT-PCR Primers

Supplementary References

[1] L. De Bolle, J. Van Loon, E. De Clercq, and L. Naesens, Quantitative analysis of human herpesvirus 6 cell tropism. J Med Virol 75 (2005) 76-85.

[2] E.L. Sonnhammer, G. von Heijne, and A. Krogh, A hidden Markov model for predicting transmembrane helices in protein sequences. Proc Int Conf Intell Syst Mol Biol 6 (1998) 175-82.
